# African Americans’ Culturally Specific Approaches to the Management of Diabetes

**DOI:** 10.1177/2333393614565183

**Published:** 2015-01-21

**Authors:** Ida J. Spruill, Gayenell S. Magwood, Lynne S. Nemeth, Tiffany H. Williams

**Affiliations:** 1 Medical University of South Carolina, Charleston, South Carolina, USA

**Keywords:** African Americans, culture, diabetes, spirituality

## Abstract

Spirituality is an important multidimensional cultural resource and coping strategy used by many African Americans for managing chronic diseases such as diabetes. Yet, few studies examine meaning and interpretation of colloquial terms frequently used for coping within the context of a community culture. We designed an interpretive qualitative study to gain a deeper understanding of a colloquial phrase, “I ain’t claiming it,” used among Project SuGar research participants when discussing diabetes. Thematic analysis revealed two major themes, Acknowledgment and Denial, as coping mechanisms through an active or passive relationship with God. Sub-theme of acknowledgment was presented as front seat driver and sub-theme for denial of the disease presented as back seat driver. These meanings encompass a range of culturally specific coping strategies for self-management that health providers should consider and implement as part of providing patient-centered care to enhance better outcome strategies.

Diabetes mellitus (DM) remains a lifelong, chronic disease with challenging health and economic outcomes. Projected direct and indirect costs in the United States alone are much more than $1.6 trillion by 2031 (Fitch, Iwasaki, & Pyenson, 2010). Responsible for more deaths than breast cancer and AIDS combined, DM is the seventh leading cause of death, affecting 8.3% of all Americans, and 11.3% of adults aged 20 and older (American Diabetes Association, 2014; Centers for Disease Control and Prevention, 2011a, 2012). Type 2 diabetes mellitus (T2DM) represents 95% of all diabetes cases (Centers for Disease Control and Prevention, 2011b).

The burden of diabetes (14.7%) among African Americans is extremely worrisome because as a group, African Americans are 1.8 times more likely to develop diabetes when compared with non-Hispanic Whites, less likely to be insured, and less likely to have access to affordable, acceptable health care (Chow, Foster, Gonzalez, & McIver, 2012). Moreover, research indicates that African Americans residing in rural areas have limited resources and poor access to care (American Diabetes Association, 2014; Centers for Disease Control and Prevention, 2008, 2011a; Probst, Moore, Glover, & Samuels, 2004; Utz, 2008), and are more likely to develop serious complications leading to blindness, renal failure, and amputations.

South Carolina is a mostly rural state with 36 of its 46 counties falling outside the metropolitan area and has one of the highest rates of diabetes for adults at 13.3% of the population (Centers for Disease Control and Prevention, 2014) affecting 8.9% of non-Hispanic Whites and 14.1 % of non-Hispanic Blacks (Centers for Disease Control and Prevention, 2008, 2011b). Diabetes is the seventh leading cause of death in South Carolina, claiming more than 3,000 lives each year, and the fifth leading cause of death in African Americans (Centers for Disease Control and Prevention, 2014; South Carolina Department of Health and Environmental Control, 2013). In spite of current diabetes self-management guidelines, which reflect a more holistic style, the incidence of diabetes continues to increase especially among rural African Americans (American Diabetes Association, 2014; Centers for Disease Control and Prevention, 2008, 2011b; Loftin, Barnett, Bunn, & Sullivan, 2005; Utz, 2008).

Research studies among rural residents are increasing but can be hampered by “culture memory” (memories of research abuse to self or family) along with a history of exploitative research (Corbie-Smith, Thomas, & St. George, 2002; Holtorf, 1996). Efforts are urgently needed to understand barriers to self-management, identify culturally appropriate prevention efforts within health care delivery systems, as well as programs designed within the cultural context of the population (Corbie-Smith et al., 2002; Probst et al., 2004). Although evidence exists that lifestyle interventions in the prevention and treatment of T2DM are effective, few studies use culture and spirituality as a construct to examine meaning and interpretation of colloquial terms.

Despite a wide body of literature regarding illness and diabetes management, African Americans have not been adequately represented in research studies, especially as it relates to the impact of culture and spirituality on self-management. To this end, we designed an interpretive qualitative study using focus groups (FGs) and informant interviews to gain a deeper understanding of the meaning and interpretation of a colloquial phrase, “I ain’t claiming it,” as relates to coping and diabetes self-management among an African American sub-culture group known as *Gullahs*.

## Background

Diabetes self-management is defined as the knowledge attained and skills required for taking care of oneself, being able to manage crises, and changing one’s lifestyle successfully. Such lifestyle changes include, but are not limited to, medication management, physical activity, and dietary compliance.

Similarly, Clark (2003) reported that there are three sets of tasks that an individual must master to ensure successful management of diabetes: (a) must be knowledgeable about the condition, the biological cause of diabetes; (b) must be able to perform activities to manage the diabetes; and (c) must apply skills essential to sustain adequate psychological functioning, such as self-efficacy and external locus of control, thus taking responsibility for managing their own diabetes (Clark, 2003). To be effective, education and interventions that facilitate self-management must be closely linked to the cultural beliefs and values of the population (Clark, 2003; Johnson-Spruill & Riegel, 2008; Mensing et al., 2006; Polzer & Miles, 2005, 2007).

The majority of people with diabetes in South Carolina live in rural areas and receive diabetes care from physicians in small group practices, and they rarely receive comprehensive diabetes self-care management education (DSME; Johnson-Spruill & Riegel, 2008). Moreover, as rural residents, they often face additional problems of multiple illnesses, travel distance from health care, limited financial resources, the complexity of medication regimens for diabetes self-care management, a lack of knowledge, and the stigma or shame of having the disease (Siminerio, Piatt, & Zgibor, 2005; Utz, 2008). Because diabetes is one of the most challenging chronic diseases to manage, researchers have developed an increased interest in self-management behaviors among African Americans, yet most studies do not include African Americans, especially rural African Americans.

## Rural African Americans and Diabetes

Utz et al.’s (2006) findings suggested that addressing health disparities in diabetes care requires that providers tailor care to the lives and understanding of their patients, and that diabetes education should be culturally tailored to recipients. Most importantly, the unique life experiences of rural African Americans require diabetes education and support consistent with their needs (Johnson-Spruill & Riegel, 2008; Pollitzer, 1991; Utz, 2008; Wenzel, Utz, Steeves, Hinton, & Jones, 2005). Studies have concluded that health care providers should find a way to support patient beliefs in a holistic approach to living with chronic disease such as diabetes. In most healing traditions and through generations of healers in the early beginnings of Western medicine, concerns of the body and spirit are intertwined (R. A. Jones et al., 2006; Samuel-Hodge et al., 2000). Today, however, a growing number of studies reveal that spirituality may play a bigger role in the coping and healing process than the medical community previously thought.

## Spirituality and African Americans

Spirituality has been defined in numerous ways, including a belief in a power operating in the universe that is greater than oneself, a sense of interconnectedness with all living creatures, an awareness of the purpose and meaning of life, and the development of personal, absolute values. Spirituality can be viewed as a way to find meaning, hope, comfort, and inner peace, yet its impact on health outcomes is poorly understood. Although spirituality is often associated with religious life, many believe that personal spirituality can be developed outside religion because acts of compassion and selflessness, altruism, and the experience of inner peace are all characteristics of spirituality.

For many African Americans, spiritual beliefs and practices serve as a source of refuge and expression, and provide a framework for overcoming racial and economic conditions. Spirituality *is deeply embedded in the rich cultural heritage of African Americans and is well intertwined into all aspects of life, including beliefs about health and illness (Parks, 2003).*

Parks (2003) further speculates that the high degree of spirituality among African Americans stems from a long history of oppression and mistreatment and that spirituality is often used as coping mechanism. Understandably, some African Americans view illness and death as yet another struggle to overcome. When viewed within this context, treatment decisions may reflect a spiritual framework that emphasizes survival guided by a relationship with a higher power. For example, “I will let God guide me and help take care of me,” or “Jesus will fix it after a while” are common religious colloquialisms in the African American culture.

Coping strategies that are particularly important for survival include hope in the face of despair, joy, peace, and internal sources of resilience for overcoming illness, and oppression. The “I am not worrying about it” or “I ain’t claiming it” phrases used in the African American community are well-known practices for coping with diseases and distress. Furthermore, prayer is another coping mechanism and a practice for meaningful connection to a higher power. It is common that elders in the African American community often refer to this as “having a talk with God” (Parks, 2003).

Polzer and Miles’s (2005) research offered some additional insights into how spirituality affects the self-management of African Americans with diabetes. Using a grounded theory design, they identified three typologies: (a) relationship and responsibility—God is in background, (b) relationship and responsibility—God is in forefront, and (c) relationship and relinquishing of self-management—God is healer. These typologies varied according to how participants viewed their relationship with God and the impact of this relationship on their self-management practice (Polzer & Miles, 2005, 2007).

Similarly, Harvey and Cook (2010) provided information about the role of spirituality in the self-management of chronic illness among non-rural older women. Enrolling a sample of 41 African Americans and non-Hispanic White women, they identified four categories to suggest the influence of spirituality in behavior change and disease management: (a) God’s involvement in illness management, (b) prayer as a mediator, (c) spirituality as a coping mechanism, and (d) the combination of conventional and spiritual practices.

Much of the research on African Americans and spirituality was conducted in urban areas, and even fewer studies examine how African Americans find meaning and interpretation of colloquial terms frequently used within the context of their own culture, communities, and environment. Our study was interested in the phrase, “I ain’t claiming it,” which was used by Project SuGar (PS) research participants when discussing their “sugar diabetes.” Therefore, our study re-contacted some of PS research participants to explore the meaning of “I ain’t claiming it.”

### Parent Study: Project SuGar (Sea Island Genetics African American Registry)

Project SuGar was designed as a community-based genetic research study initiated in 1995 to elucidate the genes responsible for the expression of diabetes and obesity among the Sea Islanders of South Carolina. The recruitment goal was to enroll 400 *Gullah* families affected by T2DM. *Gullahs* are defined as descendants of enslaved Africans living on the coast of South Carolina. The inclusion criteria included one affected sibling pair, one set of affected biological parents, and at least one parent still living. Between 1995 and 2003, 1,321 individuals consented to enroll in the study.

The research team consisted of five nurses who were hired from the local community and housed within the five Federally Qualified Community Health Centers. Recruitment was active with phone calls, face-to-face visits, and word of mouth. During the active recruitment phase of Project SuGar, the research team and nurses observed and recalled hearing the remark “I ain’t claiming it” from many patients when discussing their diabetes and shared observations during monthly research team meetings.

### Conceptual Framework

Exploring culture within the context of this study is important because it allows us to view how people construct and use symbols and language to interpret their world. For example, did the observed frequency of “I ain’t claiming it” have any meaning or was it just an empty expression passed down through generations? We used the construct of culture and spirituality to gain an understanding of “I ain’t claiming it.” As a broad concept, culture is defined as a social context of observable patterns of behaviors that refer to societal beliefs, symbols, values, lifestyles, language, and patterns of adaptive behaviors. We chose to use culture as a concept because we wanted to view the *Gullahs* within the context of their worldview. Viewing culture within their context allowed us to describe their behaviors and illness perception, as well as explore the meaning and use of the colloquial phrase. Understanding these behaviors provides a set of explicit and implicit guidelines that shape the view of their world (Molina & Aguirre-Molina, 1994).

We used spirituality as a construct because research indicates that spiritual practices tend to improve coping skills and social support, foster feelings of optimism and hope, promote healthy behavior, reduce feelings of depression and anxiety, and encourage a sense of relaxation (McBride, Arthur, Brooks, & Pilkington, 1998). Moreover, w*hen spirituality is viewed within an* African American context, folk beliefs along with healing provide coping strategies that are particularly important for survival. Furthermore, spiritually based beliefs and practices provide strategies for finding solutions to life problems as well as peace of mind. Yet, few studies use spirituality as a distinct construct that acknowledges individuals may have a faith in a divine being or force that provides a personal sense of meaning and life purpose that is separate from beliefs and practice of a particular religion (McBride et al., 1998).

To the best of our knowledge, this is the only study that is designed to explore the meaning of a colloquial phrase among the *Gullahs* of South Carolina through the lens of culture and spirituality.

## Design

We designed a qualitative interpretive study to gain a deeper understanding, meaning, and interpretation of the colloquial expression “I ain’t claiming it” used by both rural and urban African Americans as it relates to diabetes care and management. The participants were part of a larger study Project SuGar that actively enrolled patients from 1996 to 2004. Our current study was interested in finding meaning to a local phrase used by the Project SuGar research participants and how the phrase was used within the context of the *Gullah* culture, infusion of spirituality (Molina & Aguirre-Molina, 1994), and the frequency of use (McLean et al., 2003; Sale et al., 2009). Examining the *Gullah* culture within a historical context offers a central analytical frame for addressing African American health issues and coping strategies. Consequently, health-related research questions should include results that lead to policy development and implication, and culturally appropriate tangible interventions for the population.

### Sample Characteristics: The Gullahs

Isolated communities of African-origin people are set in conditions for strengthening and maintaining African-influenced traditions, spirituality, values, and beliefs. The *Gullahs* of South Carolina (Blacks with distinct African-origin language) are residents of South Carolina Sea Island communities. The people of this cultural sub-group, based on geographic origin and historical experiences, are descendants of enslaved Africans. They are residents of the Sea Island coastal communities encompassing nine counties (Charleston, Beaufort, Colleton, Dorchester, Jasper, Hampton, Allendale, Georgetown, and Horry) and were isolated from the mainland until the 1960s.

Due to cultural and geographical isolation, the *Gullahs* maintained more cultural African traditions (basket weaving) and less European admixture than any group of African Americans tested in the United States (Sale et al., 2009). Reports and observations by Blake (1984) in his classic work with the *Gullah* population of South Carolina noted that the *Gullah* people have their own way of viewing the world, coping and “making do.” Their health belief system for coping is derived from racial and cultural experiences (hope in the face of despair). Blake (1984) documented that the Sea Island population understands the many natural processes of nature and live in an area where the people are very sensitive to the weather, agriculture, and tidal waves. “They use this knowledge of nature to guide their families in meaningful ways and approach health care through a frame of reference that assumes nature has its own processes” (Blake, 1984, p. 36).

The Sea Islanders believe that “we must understand nature, become a part of it, and not try to master it,” but let God do his work. Thus, among the *Gullahs*, taking the “needle” is viewed as unnatural to them, but using prayer for coping and drinking teas for ailment may be viewed as natural. However, Blake (1984, pp. 55-62) cautions that when approaching this population, their experiences must be understood within the context of the culture.

Our approach to research among this population included immersions into the *Gullah* culture. To this end, the parent study (Project SuGar) sought guidance from the community and organized a Citizen Advisory Committee at the inception of the study in 1996 that continues to meet today. We surpassed our recruitment goal and enrolled 650 *Gullah* families (Sale et al., 2009).

## Method

We conducted an interpretive study using focus groups and key informants to find meaning and interpretation of the colloquial phrase, “I ain’t claiming it,” as it relates to diabetes self-management among an African American sub-culture known as *Gullahs*.

### Sampling Frame/Recruitment

We used a purposeful sampling frame that included self-identified African American adults with and without diabetes. We were interested in the frequency and personal meaning of the phrase, so,we selected–Project SuGar participants with diabetes or with a family history of diabetes as well as some non research participants from Project SuGar.

The Institutional Review Board (IRB) approved the study and all participants signed an informed consent. Anonymity and confidentiality were provided as no identifying information was used in the interviews or transcripts. Our participants were recruited by the research assistant (RA) using both passive recruitment strategies such as targeted individual mailings to former Project SuGar participants and active strategies such as word of mouth and recruitment flyers to non–Project SuGar participants.

### Data Collection

We collected data from five focus groups between 2008 and 2010 in rural and urban communities. Focus group sizes ranged from 5 to 12 adult African American men and women between the ages of 18 and 75 years. The focus group locations included rural churches (two), urban churches (two), and one rural African American senior citizen center.

Three informant interviews were conducted in the informants’ homes and results helped to inform the design of the focus group questions. The age range for informant interviewees was 68 to 93 years. All participants received a US $25.00 gift certificate to a supercenter retail chain store. The sessions were digitally recorded and later transcribed by a Health Insurance Portability and Accountability Act (HIPAA -approved professional transcriptionist agency. Data saturation was reached with the five focus groups.

### Interview Guide

The interview guide was designed to uncover the meaning of the phrase, “I ain’t claiming it.” It was loosely organized around topics of living with diabetes. During discussion,the participants were asked, “What does the term I ain’t claiming it” mean to you?” The discussion focused on the term, which was placed on a large poster board. The principal investigator (PI) began with the following opening remark: When discussing their diabetes in the Project SuGar research study, lots of people would say, “I ain’t claiming it.” We know that this phrase means different things to different people. Please discuss whether you have heard the term, and please share what it means to you.

### Data Analysis

A Qualitative Data Analysis (QDA) software tool using Microsoft Word® (Redmond, Washington) was used to create and format a table to manage, code, and analyze the data (LaPelle, 2004). The analysis process for this study was designed to discover rather than verify theory within contextual data. Text data were analyzed through the systematic classification process of coding and identifying themes or patterns (Creswell, 2002; Hsieh & Shannon, 2005; Strauss & Corbin, 1998). We used thematic analysis and constant comparison to identify patterns for understanding the meaning of “I ain’t claiming it.” Transcripts were read several times for accuracy and to identify themes and categories. We read line-by-line, identified discrete concepts/themes, and grouped them under labels. To address inter-rater reliability, three additional team members reviewed the coding of themes and addressed any conflicting points of view until group consensus was reached.

When new codes emerged, the coding frame changed. Using constant comparison, we were able to develop a list of coding categories relating to the phrase and categorized them during data reduction into two major themes (Hsieh & Shannon, 2005; see Table 1).

**Table 1.** Core Themes.

Theme 1. Denial of the disease	Theme 2. Acknowledgment of the disease
In relationship with God (Polzer)	In relationship with God
Sub-themes	Sub-themes
Back seat driver /active	Front seat drive/partnership
God seen as the major actor	The person with diabetes is the major actor
Assume some responsibility for care	Accepts responsibilities for self-management
Attribute positive outcome to God	Acknowledges God as a partner in care
Back seat rider/ passive	Active participant
Person with diabetes yields authority to God	God in background
God in forefront, patient in the background	

Credibility and trust of the research team were enhanced through prolonged engagement with community members, that is, field trips, attending church services, observations, interviews, or discussions with participating and non-participating rural residents. Seeking knowledge from older rural and non-rural adults, ministers, family, and other formal and informal community leaders ensured dependability (Creswell, 2002; Hsieh & Shannon, 2005; Lincoln & Guba, 1985; Strauss & Corbin, 1998).

## Results

Total number of participants was 52 (*n* = 52) and most were female and all *Gullah* and/or African Americans. Major barriers cited by focus group participants and key informants to effectively managing diabetes were lack of resources such as testing supplies, insulin, medication, and lack of knowledge regarding causation of the disease and how to prevent complications. Participants reported that having diabetes was “hard and you never get a break from it” and often invoked religion into the discussion by referring to “God as the healer.” Most expressed a lack of knowledge regarding genetics and genetic research. However, for some, they could relate genetic research to Project SuGar. When asked whether they would participate in a future genetic research study, most expressed a desire to participate in future genetic research especially if it was similar to Project SuGar.

### Frequency and Use of the Term

In terms of frequency, almost all participants (*n* = 48) reported hearing the term and offered some type of meaning or interpretation. Others reported that they use it or said that it is overused, while some participants (*n* = 4) did not verbally respond to the questions but nodded their heads in affirmation during group discussions. During the discussion about the frequency of the term, one participant stated, “Sometimes people will say those things but they don’t mean it from the heart. They say it to you to see what you are going say about it.”

### Themes From Analysis

Four themes emerged—acceptance, acknowledgment, denial, and passive. The research team collapsed the themes into the two major core themes described below: (a) acknowledgment of the disease and (b) denial of the disease. Two sub-themes were identified from the major themes with universal colloquial connotations: (a) acknowledgment/front seat driver and (b) denial/back seat rider. Both terms, *front driver* and *back seat rider*, were used by the participants during the discussion when describing self-management of diabetes. For example, when discussing controlling diabetes, one participant stated, “When you are in control of your diabetes, you are a front seat driver, and when you do not know how to manage it, you take a back seat.”

## Discussion

Using qualitative data, we explored the meaning and frequency of a colloquial term, “I ain’t claiming it,” used among the Sea Island population when discussing “sugar” diabetes. Our findings were similar to Polzer and Miles’s (2007) in that our themes acknowledged both a partnership and a relationship with God but presented themselves as somewhat of a paradox because both acknowledgment and denial can be viewed as coping mechanisms. For example, back seat drivers can acknowledge an active relationship with God when they self-medicate because they combine practice with spirituality. One participant noted, “You claiming sickness on yourself. I ain’t claiming the worrying. When you claim it, you speaking it into existence.” Similarly, a front seat driver acknowledges an active partnership with God and assumes responsibility for self-management. “I claim it cause I want to do the right thing.” “I pray to God to help me manage my sugar.”

When viewed within the cultural and spiritual context, personal views on illness will help to define a personal relationship with God. One minister who participated in the focus group offered the following:

“I think it can be positive or negative. I think it is negative when I know something is wrong with me and I say I’m not going to claim it as an excuse not to deal with it. There are certain realities that I have to deal with. On the other hand I think it can be positive when we believe and trust in God and we don’t allow things to worry us. I’m not going to claim the worrying of something. I’m not going to claim that. I think that is good. So I think it depends on how the person is using the terminology.”

Both themes/sub-themes are fueled by biblical origin with strong infusion of faith, which seems to be adaptive at first. For example, “if I put my trust in God, he will heal me, or I pray to God to show me the way.” Similarly, a quotation from the bible, “death and life are in the power of the tongue,” and “those who love it will eat its fruits,” Proverbs 18:21 (p. 624) and Matthew 21:22 (p. 904) (American Bible Society, KJV 1999), respectively, and “whatever you ask in prayer, you will receive if you have faith.” Oftentimes, during the discussions, participants’ statements implied a spiritual relationship with God. Remarks such as “I put it in God’s hand” may persist as denial, indicating a passive relationship with God, suggesting passive behavior and correlating with a lack of involvement in diabetes self-management.

### Acknowledgment of the Disease: Front Seat Driver

We categorize acknowledgment of the disease as a front seat driver because the term indicates control or active behavior for steering a vehicle, personal involvement, and a position of power or dominance. Participants who are front seat drivers have an active partnership with God and acknowledge their disease. They take responsibility and assume more of a partner’s role with God in the background as a helper. Front seat drivers chose to sit in the front seat as equal partners in their care and have internal locus of control. They perceive their partnership with God as a source of support in their self-management practices. For example, in acknowledging the healing power and presence of God, one participant noted, “You gave me the knowledge to take care of myself.”

They trust the role of the provider because he or she was made in God’s image as “God gave them the knowledge.” Another participant noted,

“So we’ve got to acknowledge that we are Christian, spiritual, yeah God gave us these people that we need to go to for better health, but if you just look to God and not look to each other then you are going to fail.”

Comments from some participants suggest acknowledgment in that health care providers are part of God’s creations and should be trusted as healers. A comment from one participant indicated “God works through doctors.”

### Denial of the Disease: Back Seat Driver

Although back seat drivers sit in the back and give direction to the driver, they too have a relationship with God. Unlike front seat drivers, a back seat driver is a passenger who is not in control of the vehicle. He or she may not be able to drive,do not know causation of the disease does not trust own self to drive, and/or is uncomfortable with the skills needed to manage the disease. Similar to comments from Clark (2003), back seat drivers lack all three sets of tasks needed for self-management.

A back seat driver chooses to ride in the back seat and use his or her relationship with God to help manage his or her illness. They attribute their success with disease management to the work of the Lord. “God made me in his image and I cannot fail,” because “God is a healer.” However, a back seat rider is waiting for God to take control and God is acknowledged as the healer, or the fixer. In some instances, the back seat driver will become a passive rider and relinquish all care to God. For example, one participant said, “I am not going to claim it because I know that He is going to help me to overcome this thing through prayer.” This was also evident in the individual interviews, “God is the healer.”

Our participants’ comments were similar to work by other researchers who also reported that some African Americans view the physicians as the healer or the instrument through whom God administers healing (Abrums, 2000; Conner & Eller, 2004; Davis & Magilvy, 2000; Harvey & Cook, 2010; McAuley, Pecchioni, & Grant, 2000; Polzer & Miles, 2005).

However, unlike previous research, we discovered a level of personal participation in self-management among the participants who were categorized as denial back seat drivers. Although some indicated that they might not follow recommendations from their providers, they chose to participate in their care by using prayer and alternative medicines to treat their diabetes.

We noted very few participants who were passive riders and relinquished total control of their care to God. In fact, only two individuals (one rural/one urban) exhibited passive rider behaviors, with statements such as “God will cure my sugar,” “I do not believe it,” “I will not accept or acknowledge it,” “and I do not have sugar.” It is important to note that our participants’ comments on healing supported results from McAuley and colleagues’ (2000) statement that “there is a set of religious truths that do not require further investigation or analysis, and in a manner indicating that religious belief permeated their lives.”

Although both Polzer and Miles (2007) and Harvey and Cook (2010) presented more than two themes/categories, our study chose to interpret the term within the participants’ view or sense of their world. If we wish to understand culture, language, and human behavior, we must explore how people define, explain, or react to what they encounter in their environment because human beings act toward things on the basis of the meaning they have or learn from generations.

According to Blumer (1973), they sometimes ascribe their own meaning or accept what was passed down. “I ain’t claiming it” was passed down through the years and the meaning of the term varies among the users, and based on how it was passed down, it can invoke either positive or negative behaviors. You accept the disease and work with God as a partner, ask for help, or you relinquish control and/or care and view God as the fixer. At the foundation of African Americans’ worldview are values and principles such as belief in God and honoring the continuity of life by respecting kinship bonds, ancestors, and a communal way of life. Moreover, African Americans possess shared beliefs, values, and principles that may underlie and shape behaviors that are found in social relations (Blumer, 1973; Conner & Eller, 2004; Davis & Magilvy, 2000; J. Jones, 1991).

When viewed within the African culture context, especially the *Gullah* population, relationships and responsibilities are family-oriented and communal. Respect is granted to elders and family mores have been intact for generations. In the *Gullah* culture, family members are socialized to give meaning to symbols such as sweet grass baskets, and their *Gullah* language, a creole language that has many symbols and meanings passed down through generations. Their sense of the world and spiritual beliefs may change over the years, or lose their interaction, but remnants of the language will remain with others in the community.

Children are born hearing phrases and learn to give meaning to them based on interaction with their elders. So when they hear “I ain’t claiming it” from their elders, and view their behaviors (passive or active), their responses later in life may mirror or model what was passed down. They may internalize, ascribe their own meaning, or refuse to accept what was passed down. Thus, phrases such as “I ain’t claiming it” may be lost. Nonetheless, these meanings are modified through an interpretive process, and interpretation of the meaning will guide and determine action that may result in tangible resources and/or improved diabetes self-management.

### Limitations

This study was conducted among African Americans residing in South Carolina and may not be generalizable to other rural or urban African American populations. Some of the questions in the instrument contained loaded barrel questions. For example, the researcher could have asked, or added for clarity, “If you heard the term, yes or no? If yes, do you use it? Describe what it means to you. If no, have you heard other people use it? Do you use it? Why do you use it?” Future research questions could include a comparative analysis of the meaning between rural and urban African Americans. In addition, future research should include the impact of front seat driver and back seat driver behavior on diabetes self-management, glycemic control, and incidence of complications.

## Conclusion

Isolated from the mainland for many years, the *Gullah* population maintains many African-influenced styles and practices, which were blended into Christian religion (J. Jones, 1991; Pollitzer, 1991). Spirituality is present in every aspect of life. Providers can view these local definitions and meanings passed down over the years as culturally specific coping behaviors (Abrums, 2000; Conner & Eller, 2004; Davis & Magilvy, 2000; McAuley et al., 2000).

Acknowledging the support of patients’ spiritual beliefs is more appropriate than ignoring it especially if it improves self-management skills. Although health care providers may find it unethical or inappropriate to discuss spirituality or religious issues, they could enhance delivery of culturally sensitive care by understanding that these phrases may be used by participants as a coping mechanism and can help to improve health outcomes and reduce complications.

Although many are still using “I ain’t claiming it,” the phrase may have lost its connotation to some and may be cited without meaning. Nonetheless, because it is embedded in the culture, embraced by many, spoken across disciplines and illness, these culturally appropriate strategies should not be used to stigmatize populations, but instead used to improve diabetes self-management and health outcomes.

## References

[bibr1-2333393614565183] Abrums M. (2000). “Jesus will fix it after awhile”: Meanings and health. Social Science & Medicine, 50, 89–105. doi:10.1016/S0277-9536(99)00277-4 10622697

[bibr2-2333393614565183] American Bible Society. (1999). The Holy Bible: Containing the Old and New Testaments: King James version. New York: Author.

[bibr3-2333393614565183] American Diabetes Association. (2014). Statistics about diabetes. Retrieved from http://www.diabetes.org/diabetes-basics/statistics/

[bibr4-2333393614565183] Blake H. (1984). “Doctor can’t do me no good”: Social concomitants of health care attitudes and practices among elderly blacks in isolated rural populations. In Watson W. H. (Ed.), Black Folk Medicine (pp. 55-62). New Brunswick: Transaction Books.

[bibr5-2333393614565183] Blumer H. (1973). Notes on symbolic interactionism. American Sociological Review, 38, 797–800.

[bibr6-2333393614565183] Centers for Disease Control and Prevention. (2008). CDC newsroom press release June 24, 2008. Retrieved from http://www.cdc.gov/media/pressrel/2008/r080624.htm

[bibr7-2333393614565183] Centers for Disease Control and Prevention. (2011a). Diabetes data and trends. Retrieved from http://apps.nccd.cdc.gov/ddtstrs/default.aspx

[bibr8-2333393614565183] Centers for Disease Control and Prevention. (2011b). National diabetes fact sheet: National estimates and general information on diabetes and prediabetes in the United States. Retrieved from http://www.cdc.gov/brfss/index.htm

[bibr9-2333393614565183] Centers for Disease Control and Prevention. (2012). Diabetes report card 2012. Retrieved from http://www.cdc.gov/diabetes/pubs/reportcard.htm

[bibr10-2333393614565183] Centers for Disease Control and Prevention. (2014). National diabetes statistics report: Estimates of diabetes and its burden in the United States, 2014. Retrieved from http://www.cdc.gov/diabetes/pubs/estimates14.htm

[bibr11-2333393614565183] Chow E. Foster H. Gonzalez V. McIver L. (2012). The disparate impact of diabetes on racial/ethnic minority populations. Clinical Diabetes, 30, 130–133. doi:10.2337/diaclin.30.3.130

[bibr12-2333393614565183] Clark N. M. (2003). Management of chronic disease by patients. Annual Review of Public Health, 24, 289–313. doi:10.1146/annurev.publhealth.24.100901.141021 12415147

[bibr13-2333393614565183] Conner N. E. Eller L. S. (2004). Spiritual perspectives, needs, and nursing interventions of Christian African-Americans. Journal of Advanced Nursing, 46, 624–632. doi:10.1111/j.1365-2648.2004.03053.x 15154903

[bibr14-2333393614565183] Corbie-Smith G. Thomas S. B. St. George D. M. (2002). Distrust, race, and research. Archives of Internal Medicine, 162, 2458–2463. doi:10.1001/archinte.162.21.2458 12437405

[bibr15-2333393614565183] Creswell J. (2002). Educational research: Planning, conducting and evaluating qualitative and quantitative research. Saddle River, NJ: Prentice Hall.

[bibr16-2333393614565183] Davis R. Magilvy J. K. (2000). Quiet pride: The experience of chronic illness by rural older adults. Journal of Nursing Scholarship, 32, 385–390. doi:10.1111/j.1547-5069.2000.00385.x 11140203

[bibr17-2333393614565183] Fitch K. Iwasaki K. Pyenson B. (2010). Improved management can help reduce economic burden of Diabetes. New York: Milliman.

[bibr18-2333393614565183] Harvey I. S. Cook L. (2010). Exploring the role of spirituality in self-management practices among older African-American and non-Hispanic White women with chronic conditions. Chronic Illness, 6, 111–124. doi:10.1177/1742395309350228 20444763

[bibr19-2333393614565183] Holtorf C. J. (1996). Towards a chronology of megaliths: Understanding monumental time and cultural memory. Journal of European Archaeology, 4, 119–152. doi:10.1179/096576696800688051

[bibr20-2333393614565183] Hsieh H. F. Shannon S. E. (2005). Three approaches to qualitative content analysis. Qualitative Health Research, 15, 1277–1288. doi:10.1177/1049732305276687 16204405

[bibr21-2333393614565183] Johnson-Spruill I. Riegel B. (2008). The quality of diabetes care to Gullah families of South Carolina. Journal of National Black Nurses Association, 19(2), 20–27. PMC2712444 19397050

[bibr22-2333393614565183] Jones J. (1991). Racism: A culture analysis of the problem (3rd ed.). Berkeley, CA: Black Psychology.

[bibr23-2333393614565183] Jones R. A. Utz S. Wenzel J. Steeves R. Hinton I. Andrews D.. . .Oliver N. (2006). Use of complementary and alternative therapies by rural African Americans with type 2 diabetes. Alternative Therapies in Health and Medicine, 12(5), 34–38. PMC3665110 17017753

[bibr24-2333393614565183] LaPelle N. (2004). Simplifying qualitative data analysis using general purpose software tools. Field Methods, 16, 85–108. doi:10.1177/1525822x03259227

[bibr25-2333393614565183] Lincoln Y. Guba E. (1985). Naturalistic inquiry. Newbury Park, CA: SAGE.

[bibr26-2333393614565183] Loftin W. A. Barnett S. K. Bunn P. S. Sullivan P. (2005). Recruitment and retention of rural African Americans in diabetes research: Lessons learned. Diabetes Educator, 31, 251–259. doi:10.1177/0145721705275517 15797854

[bibr27-2333393614565183] McAuley W. J. Pecchioni L. Grant J. A. (2000). Personal accounts of the role of God in health and illness among older rural African American and White residents. Journal of Cross Cultural Gerontology, 15, 13–35. doi:10.1023/A:1006745709687 14618008

[bibr28-2333393614565183] McBride J. L. Arthur G. Brooks R. Pilkington L. (1998). The relationship between a patient’s spirituality and health experiences. Family Medicine, 30(2), 122–126. 9494803

[bibr29-2333393614565183] McLean D. C. Jr. Spruill I. Gevao S. Morrison E. Y. Bernard O. S. Argyropoulos G. Garvey W. T. (2003). Three novel mtDNA restriction site polymorphisms allow exploration of population affinities of African Americans. Human Biology, 75(2), 147–161. 12943155 10.1353/hub.2003.0035

[bibr30-2333393614565183] Mensing C. Boucher J. Cypress M. Weinger K. Mulcahy K. Barta P.. . .Adams C. (2006). National standards for diabetes self-management education. Diabetes Care, 29(Suppl. 1), S78–S85. doi:10.1177/0145721712455997 16373939

[bibr31-2333393614565183] Molina C. W. Aguirre-Molina M. (1994). The influence of culture class and environment on health care. (Eds)In Latino health in the US: A growing challenge (pp. 23–44).Washington, DC: American Public Health Association, University of Texas.

[bibr32-2333393614565183] Parks F. M. (2003). The role of African American folk beliefs in the modern therapeutic process. Clinical Psychology: Science and Practice, 10, 456–467. doi:10.1093/clipsy.bpg046

[bibr33-2333393614565183] Pollitzer W. S. (1991). The Gullah people and their African heritage. Athens: University of Georgia Press.

[bibr34-2333393614565183] Polzer R. Miles M. S. (2005). Spirituality and self-management of diabetes in African Americans. Journal of Holistic Nursing, 23, 230–250; discussion 251–234; quiz 226–237. doi:10.1177/0898010105276179 15883469

[bibr35-2333393614565183] Polzer R. Miles M. S. (2007). Spirituality in African Americans with diabetes: Self-management through a relationship with God. Qualitative Health Research, 17, 176–188. doi:10.1177/1049732306297750 17220389

[bibr36-2333393614565183] Probst J. C. Moore C. G. Glover S. H. Samuels M. E. (2004). Person and place: The compounding effects of race/ethnicity and rurality on health. American Journal of Public Health, 94, 1695–1703. doi:10.2105/AJPH.94.10.1695 15451735 PMC1448519

[bibr37-2333393614565183] Sale M. M. Lu L. Spruill I. J. Fernandes J. K. Lok K. H. Divers J.. . .Garvey W. T. (2009). Genome-wide linkage scan in Gullah-speaking African American families with type 2 diabetes: The Sea Islands Genetic African American Registry (Project SuGar). Diabetes, 58, 260–267. doi:10.2337/db08-0198 18835935 PMC2606883

[bibr38-2333393614565183] Samuel-Hodge C. D. Headen S. W. Skelly A. H. Ingram A. F. Keyserling T. C. Jackson E. J.. . .Elasy T. A. (2000). Influences on day-to-day self-management of type 2 diabetes among African-American women: Spirituality, the multi-caregiver role, and other social context factors. Diabetes Care, 23, 928–933. doi:10.2337/diacare.23.7.928 10895842

[bibr39-2333393614565183] Siminerio L. M. Piatt G. Zgibor J. C. (2005). Implementing the chronic care model for improvements in diabetes care and education in a rural primary care practice. Diabetes Educator, 31, 225–234. doi:10.1177/0145721705275325 15797851

[bibr40-2333393614565183] South Carolina Department of Health and Environmental Control. (2013). Public health data, maps and reports. Retrieved from http://www.scdhec.gov/Health/SCPublicHealthStatisicsMaps/

[bibr41-2333393614565183] Strauss A. Corbin J. (1998). Basics of qualitative research: Grounded theory procedures and techniques (2nd ed.). Thousand Oaks, CA: SAGE.

[bibr42-2333393614565183] Utz S. (2008). Diabetes care among rural African Americans. In Annual review of nursing research (pp. 3–39). New York: Springer Publishing. 18709745

[bibr43-2333393614565183] Utz S. Steeves R. H. Wenzel J. Hinton I. Jones R. A. Andrews D.. . .Oliver M. N. (2006). “Working hard with it”: Self-management of type 2 diabetes by rural African Americans. Family & Community Health, 29, 195–205. doi:10.1097/00003727-200607000-00006 16775469

[bibr44-2333393614565183] Wenzel J. Utz S. W. Steeves R. Hinton I. Jones R. A. (2005). “Plenty of sickness”: Descriptions by African Americans living in rural areas with type 2 diabetes. Diabetes Educator, 31, 98–107. doi:10.1177/0145721704273242 15779251

